# Supplementary material for: A Likelihood Approach for Real-Time Calibration of Stochastic Compartmental Epidemic Models
Source: PLoS Comput Biol. 2017 Jan 17;13(1):e1005257. doi: 10.1371/journal.pcbi.1005257 (PMC5240920; doi:10.1371/journal.pcbi.1005257)
Supplement: S1 File — (TAR.GZ) [file pcbi.1005257.s014.tar.gz › HSPH_Online-SI-Revision/MSS10/n10-extreme/n10-extreme_NEW_table0.pdf]

1	R0	{2.68692, 12.5884, 1.}	{2.24278, 9.59251, 0.34}	{{2.22376, 9.35432, 0.}, {2.38758, 11.1331, 1.}}	{16.5295, 23.7988, 90.}	inf	2477.	1841.94	{1765.25, 1960.51}	25.6382	0.129206	newly	1264.	1215.35	{1152., 1264.}	4.08307	0.0183336	Reff	0.771415	0.785789	{0.772488, 0.801071}	1.96575	ar	9089.	8523.59	{8400., 8705.}	6.22082
2	R0	{1.79802, 12.5412, 1.}	{1.61059, 8.77952, 3.35}	{{1.6082, 8.77064, 3.}, {1.61188, 8.7843, 4.}}	{10.4242, 29.9946, 235.}	inf	1208.	806.322	{758.441, 851.288}	33.2515	0.17597	newly	678.	625.15	{582., 669.}	7.99263	0.0366908	Reff	1.00509	0.965014	{0.956701, 0.972191}	3.98762	ar	7412.	6432.1	{6300., 6561.}	13.2205
3	R0	{2.6174, 10.1105, 0.1}	{2.6821, 10.2195, 0.35}	{{2.52681, 9.219, 0.}, {2.70795, 10.355, 2.}}	{4.1189, 4.30354, 408.}	inf	2244.	2311.31	{2114.68, 2408.2}	5.61805	0.0241607	newly	1325.	1378.13	{1330., 1423.}	4.27245	0.0180648	Reff	0.662726	0.659814	{0.646268, 0.674262}	1.42785	ar	9109.	9126.76	{8922., 9209.}	0.924141
4	R0	{2.27532, 14.3149, 0.1}	{1.88006, 10.2873, 1.96}	{{1.87584, 10.2502, 2.}, {1.87584, 10.2502, 2.}}	{17.3715, 28.1357, 1860.}	inf	1860.	1188.02	{1139.72, 1225.02}	36.1281	0.195091	newly	862.	743.26	{701., 786.}	13.7749	0.0647768	Reff	0.820025	0.803821	{0.795627, 0.810968}	1.97605	ar	8478.	7584.46	{7472., 7697.}	10.5395
5	R0	{2.96649, 18.0674, 2.}	{2.6816, 15.4607, 2.}	{{2.48103, 13.7658, 2.}, {2.8833, 17.1847, 2.}}	{9.60372, 14.4276, 0.}	inf	2876.	2395.27	{2116.58, 2687.9}	16.7153	0.0830059	newly	1043.	1006.39	{966., 1050.}	4.07191	0.0181887	Reff	0.663011	0.72172	{0.697357, 0.74252}	8.85504	ar	9476.	9101.76	{8853., 9356.}	3.94935
6	R0	{2.94778, 16.0002, 0.1}	{2.49774, 13.1452, 1.35}	{{2.15677, 10.1363, 0.}, {2.688, 14.8279, 2.}}	{15.2671, 17.8432, 1306.}	inf	2859.	2338.43	{1758.04, 2680.36}	18.2082	0.0944354	newly	1175.	1158.06	{1106., 1196.}	2.50553	0.0110594	Reff	0.814766	0.810335	{0.7895, 0.834336}	1.82595	ar	9321.	8894.15	{8334., 9228.}	4.57944
7	R0	{2.17562, 11.7184, 1.}	{2.37719, 12.0416, 2.25}	{{1.39162, 1.89883, 0.}, {2.78007, 16.1081, 3.}}	{29.6877, 49.7513, 163.}	inf	1758.	2108.02	{291.048, 2893.83}	67.2966	0.364187	newly	1027.	1077.86	{683., 1234.}	20.4752	0.0948805	Reff	0.998827	0.937564	{0.841335, 0.982712}	6.13351	ar	8345.	8034.35	{4910., 9298.}	19.087
8	R0	{2.7904, 17.8966, 2.}	{2.04197, 10.5854, 1.}	{{1.95791, 9.82151, 1.}, {2.47514, 14.5291, 1.}}	{26.8218, 40.8526, 50.}	inf	2748.	1541.97	{1329.36, 2351.8}	43.8877	0.261066	newly	978.	955.49	{885., 1093.}	6.32413	0.0277145	Reff	0.871163	0.876609	{0.863527, 0.888027}	1.08713	ar	9285.	7990.19	{7697., 8929.}	13.9452
9	R0	{2.51574, 16.376, 1.}	{2.75925, 18.2439, 2.}	{{2.76849, 18.33, 2.}, {2.76849, 18.33, 2.}}	{9.67926, 11.5027, 100.}	inf	2361.	2618.91	{2581.23, 2671.87}	11.2689	0.0463396	newly	932.	962.68	{917., 991.}	3.83476	0.0162844	Reff	0.84076	0.850643	{0.838822, 0.864235}	1.33919	ar	8937.	9203.1	{9157., 9261.}	2.98378
10	R0	{2.56405, 18.1787, 2.}	{2.32601, 15.7669, 3.}	{{1.95639, 11.2959, 3.}, {2.46976, 17.5057, 3.}}	{9.28356, 13.267, 50.}	inf	2387.	1985.26	{1456.53, 2231.76}	16.8305	0.086563	newly	979.	867.57	{829., 907.}	11.382	0.052741	Reff	0.996903	0.990421	{0.939329, 1.02169}	2.64912	ar	9005.	8554.8	{7808., 8885.}	4.99944
11	R0	{2.475, 17.1709, 1.}	{1.52365, 4.72873, 0.24}	{{1.47488, 3.83841, 0.}, {1.67405, 7.28757, 1.}}	{38.4385, 72.4608, 78.}	inf	2191.	552.034	{398.088, 878.613}	74.8045	0.618312	newly	841.	693.79	{592., 785.}	17.5042	0.0860136	Reff	0.935303	0.824077	{0.803262, 0.855681}	11.8919	ar	8870.	5913.56	{5467., 6752.}	33.3308
12	R0	{2.14974, 11.8619, 0.1}	{2.0179, 10.6445, 0.06}	{{2.02235, 10.6804, 0.}, {2.02235, 10.6804, 0.}}	{6.13303, 10.2631, 148.}	inf	1788.	1644.1	{1571.23, 1698.89}	8.04803	0.0368034	newly	1021.	1062.48	{1015., 1108.}	4.59158	0.0193911	Reff	1.0409	1.006	{0.99618, 1.01561}	3.35329	ar	8414.	8055.28	{7953., 8147.}	4.26337
13	R0	{2.20385, 14.4767, 0.1}	{1.74261, 8.47294, 1.21}	{{1.67405, 7.28757, 1.}, {1.77826, 9.06807, 2.}}	{20.9286, 41.4719, 1110.}	inf	1745.	966.75	{786.43, 1096.42}	44.5988	0.260328	newly	848.	722.36	{662., 777.}	14.816	0.0704027	Reff	0.831733	0.792716	{0.784103, 0.801773}	4.69106	ar	8347.	7102.92	{6754., 7372.}	14.9045
14	R0	{2.35671, 13.8357, 1.}	{1.76435, 7.38554, 3.07}	{{1.739, 7.10524, 3.}, {1.75445, 7.55154, 3.}}	{25.9821, 47.6064, 207.}	inf	2049.	971.805	{877.45, 995.595}	53.8039	0.335875	newly	946.	817.03	{760., 863.}	14.0095	0.0659882	Reff	0.81118	0.793523	{0.784433, 0.803268}	2.17825	ar	8719.	7140.59	{6958., 7245.}	18.3508
15	R0	{1.91368, 10.1082, 1.}	{2.35866, 16.9062, 2.}	{{2.35866, 16.9062, 2.}, {2.35866, 16.9062, 2.}}	{23.2527, 67.2526, 100.}	inf	1215.	2172.	{2127.57, 2215.96}	78.7652	0.252225	newly	838.	863.78	{833., 899.}	3.54654	0.0150398	Reff	0.888904	0.869875	{0.859505, 0.880334}	2.16961	ar	7486.	8757.25	{8697., 8814.}	16.9817
16	R0	{2.48727, 14.882, 0.1}	{2.93371, 17.1903, 1.}	{{2.93371, 17.1903, 1.}, {2.93371, 17.1903, 1.}}	{17.9488, 15.5107, 900.}	inf	2351.	2905.15	{2859.07, 2948.16}	23.5709	0.091885	newly	1082.	1145.17	{1105., 1183.}	5.86414	0.0246095	Reff	0.913574	0.914979	{0.902222, 0.928397}	0.95511	ar	8894.	9345.44	{9306., 9387.}	5.07578
17	R0	{2.46795, 13.1915, 0.1}	{1.70566, 6.19284, 1.98}	{{1.69756, 6.15907, 2.}, {1.69756, 6.15907, 2.}}	{30.8875, 53.0543, 1884.}	inf	2177.	950.006	{864.582, 1028.53}	56.3617	0.363493	newly	1116.	966.12	{897., 1041.}	13.7832	0.0650582	Reff	0.942263	0.859781	{0.84631, 0.871022}	8.75359	ar	8796.	6928.78	{6728., 7145.}	21.2281
18	R0	{2.96603, 16.7543, 0.1}	{2.50617, 13.9604, 1.34}	{{1.93419, 8.56054, 0.}, {2.85582, 17.2484, 2.}}	{15.5043, 19.8022, 1306.}	inf	2830.	2171.46	{1219.06, 2758.35}	23.27	0.142682	newly	1119.	995.82	{896., 1070.}	11.008	0.0516149	Reff	0.741508	0.761271	{0.732943, 0.797895}	3.21875	ar	9394.	8714.96	{7675., 9337.}	7.22844
19	R0	{2.25216, 9.90465, 0.1}	{2.6709, 12.3901, 0.92}	{{2.71225, 12.7047, 1.}, {2.71225, 12.7047, 1.}}	{20.3991, 28.613, 836.}	inf	1991.	2554.93	{2550.93, 2674.01}	32.4182	0.127004	newly	1350.	1379.46	{1335., 1430.}	3.74519	0.0162102	Reff	0.932845	0.949542	{0.932449, 0.966178}	2.12568	ar	8631.	9072.64	{9096., 9205.}	6.59599
20	R0	{2.70837, 18.1923, 0.1}	{2.17722, 12.0888, 1.52}	{{1.75445, 7.55154, 1.}, {2.40085, 15.146, 3.}}	{19.8368, 33.9456, 1420.}	inf	2671.	1851.01	{1092.9, 2252.8}	30.7056	0.171398	newly	986.	1015.84	{903., 1104.}	7.09533	0.0301407	Reff	0.903241	0.895807	{0.8704, 0.913911}	1.50924	ar	9183.	8334.56	{7276., 8849.}	9.29326
21	R0	{2.30288, 15.5877, 1.}	{2.11292, 12.9719, 0.}	{{2.11292, 12.9719, 0.}, {2.11292, 12.9719, 0.}}	{8.24883, 16.7811, 100.}	inf	2049.	1560.44	{1523.12, 1601.69}	23.8439	0.118377	newly	836.	803.29	{778., 832.}	4.25	0.0189529	Reff	0.842854	0.876557	{0.867707, 0.884053}	3.99872	ar	8674.	8181.74	{8089., 8260.}	5.67512
22	R0	{2.82208, 10.7544, 0.1}	{2.45487, 8.95337, 1.66}	{{2.20704, 7.29612, 0.}, {2.5684, 9.75224, 3.}}	{13.0122, 16.7469, 1582.}	inf	2783.	2140.36	{1788.98, 2420.42}	23.0918	0.117084	newly	1622.	1491.1	{1424., 1545.}	8.07028	0.0367354	Reff	0.73233	0.790723	{0.769516, 0.807137}	7.97363	ar	9333.	8829.68	{8438., 9090.}	5.39291
23	R0	{1.69923, 10.1366, 1.}	{1.57017, 6.89016, 1.4}	{{1.44762, 4.00571, 0.}, {1.62191, 8.12461, 2.}}	{7.59545, 32.027, 100.}	inf	1080.	706.734	{360.484, 888.134}	34.5617	0.209522	newly	667.	660.36	{558., 722.}	6.93553	0.0311185	Reff	0.906029	0.894177	{0.867567, 0.910418}	1.52962	ar	7055.	6164.6	{5244., 6670.}	12.6208
24	R0	{2.64944, 19.054, 1.}	{2.76795, 19.6041, 1.}	{{2.76795, 19.6041, 1.}, {2.76795, 19.6041, 1.}}	{4.47298, 2.88729, 0.}	inf	2322.	2517.92	{2477.29, 2549.57}	8.43771	0.035153	newly	778.	839.91	{810., 871.}	7.95758	0.0330784	Reff	0.697333	0.674294	{0.664017, 0.684068}	3.30389	ar	9147.	9234.49	{9189., 9280.}	0.956488
25	R0	{2.53191, 10.9214, 0.1}	{1.89497, 6.21161, 1.63}	{{1.81768, 5.68021, 1.}, {2.19024, 8.25876, 4.}}	{25.1567, 43.1245, 1530.}	inf	2411.	1208.87	{1016.25, 1728.43}	49.8604	0.307567	newly	1327.	1161.9	{1050., 1292.}	12.5803	0.0595162	Reff	0.740077	0.778601	{0.764969, 0.791434}	5.20539	ar	9009.	7622.16	{7307., 8417.}	15.3939
26	R0	{1.89155, 6.76944, 0.1}	{1.84602, 6.69806, 0.35}	{{1.74092, 5.83416, 0.}, {1.75359, 5.92488, 1.}}	{11.8016, 23.6472, 412.}	inf	989.	1110.36	{884.458, 1037.78}	22.3487	0.0672753	newly	913.	998.43	{912., 1105.}	9.72289	0.0389996	Reff	0.863871	0.804154	{0.792375, 0.815246}	6.9127	ar	7348.	7290.52	{6940., 7310.}	5.89684
27	R0	{2.63149, 15.6979, 0.1}	{2.64385, 14.6877, 0.71}	{{1.93419, 8.56054, 0.}, {2.93371, 17.1903, 1.}}	{15.8386, 19.9354, 668.}	inf	2448.	2520.09	{1366.53, 3014.46}	27.9447	0.131755	newly	1086.	1141.6	{1056., 1200.}	6.36096	0.0266863	Reff	0.945758	0.898464	{0.887064, 0.910555}	5.00057	ar	9104.	8904.2	{7735., 9400.}	6.26999
28	R0	{2.85141, 12.6992, 1.}	{2.65185, 11.2069, 1.69}	{{2.19024, 8.25876, 1.}, {2.79531, 12.1249, 2.}}	{6.99854, 11.7514, 69.}	inf	2712.	2433.78	{1763.99, 2698.29}	10.3001	0.0554106	newly	1362.	1361.34	{1287., 1421.}	3.31424	0.0147172	Reff	0.727965	0.740792	{0.716774, 0.769355}	2.45468	ar	9317.	9054.73	{8440., 9296.}	2.82977
29	R0	{2.54244, 12.9085, 0.1}	{2.03307, 8.59564, 1.}	{{2.03307, 8.59564, 1.}, {2.03307, 8.59564, 1.}}	{20.0349, 33.411, 900.}	inf	2318.	1445.8	{1390.18, 1498.97}	37.6273	0.205203	newly	1154.	1061.12	{1021., 1104.}	8.04853	0.0366518	Reff	0.769342	0.79253	{0.781271, 0.803716}	3.01392	ar	9034.	8031.19	{7941., 8108.}	11.1004
30	R0	{2.10982, 11.7636, 1.}	{2.64334, 17.2462, 1.}	{{2.64334, 17.2462, 1.}, {2.64334, 17.2462, 1.}}	{25.2875, 46.6063, 0.}	inf	1626.	2431.33	{2384.05, 2476.48}	49.5281	0.174685	newly	882.	954.3	{920., 989.}	8.19728	0.0340149	Reff	0.892032	0.904729	{0.892721, 0.917049}	1.47001	ar	8224.	9066.4	{9003., 9116.}	10.2432
31	R0	{2.76312, 15.5396, 0.1}	{2.58823, 14.0705, 2.23}	{{2.58092, 13.7736, 2.}, {2.62879, 15.1183, 3.}}	{6.32932, 9.45405, 2132.}	inf	2551.	2413.42	{2329.36, 2584.01}	6.14561	0.0283084	newly	1066.	1121.82	{1083., 1153.}	5.54409	0.0233759	Reff	0.809318	0.792752	{0.761294, 0.810857}	2.26009	ar	9220.	9039.77	{8967., 9165.}	1.95889
32	R0	{2.21727, 15.5439, 0.1}	{1.71198, 8.74626, 1.02}	{{1.71337, 8.80249, 1.}, {1.71337, 8.80249, 1.}}	{22.789, 43.7319, 920.}	inf	1810.	954.781	{910.784, 998.47}	47.2497	0.27804	newly	784.	704.81	{668., 743.}	10.2538	0.0474301	Reff	0.888682	0.832566	{0.824726, 0.840395}	6.31448	ar	8359.	6992.88	{6860., 7125.}	16.3431
33	R0	{2.72912, 10.8159, 0.1}	{2.34753, 8.56429, 0.66}	{{2.26582, 8.12562, 0.}, {2.44736, 9.16315, 1.}}	{13.982, 20.8176, 656.}	inf	2166.	1592.74	{1419.64, 1780.52}	26.4664	0.135541	newly	1179.	1084.69	{1017., 1155.}	8.08567	0.0371081	Reff	0.537091	0.595203	{0.57441, 0.613454}	10.8198	ar	9196.	8684.59	{8489., 8903.}	5.56122
34	R0	{2.20928, 10.1308, 1.}	{2.32678, 10.0266, 4.}	{{2.32678, 10.0266, 4.}, {2.32678, 10.0266, 4.}}	{5.31868, 1.02815, 300.}	inf	1753.	1940.96	{1881.74, 1997.17}	10.722	0.0441298	newly	1080.	1219.19	{1181., 1259.}	12.888	0.0524905	Reff	0.754911	0.751329	{0.738106, 0.764262}	1.12615	ar	8388.	8670.52	{8605., 8736.}	3.36814
35	R0	{2.10103, 15.2673, 2.}	{2.03831, 13.1693, 3.}	{{2.03831, 13.1693, 3.}, {2.03831, 13.1693, 3.}}	{2.98541, 13.7421, 50.}	inf	1737.	1561.9	{1521.49, 1601.62}	10.0805	0.0462479	newly	782.	799.39	{764., 835.}	3.41304	0.0145588	Reff	0.894198	0.903193	{0.894908, 0.9113}	1.07741	ar	8264.	8053.62	{7957., 8135.}	2.54574
36	R0	{2.74914, 19.2775, 1.}	{2.11292, 12.9719, 0.}	{{2.11292, 12.9719, 0.}, {2.11292, 12.9719, 0.}}	{23.1425, 32.7096, 100.}	inf	2701.	1798.49	{1752.36, 1838.37}	33.414	0.176686	newly	946.	918.31	{879., 955.}	3.39218	0.0150846	Reff	0.859106	0.850981	{0.842537, 0.860506}	1.03271	ar	9301.	8319.81	{8233., 8381.}	10.5493
37	R0	{2.78161, 16.0136, 0.1}	{2.1404, 9.90151, 1.11}	{{1.78724, 6.63253, 1.}, {2.22671, 10.7276, 2.}}	{23.0518, 38.1681, 1018.}	inf	2648.	1662.89	{1001.07, 1876.02}	37.202	0.214648	newly	1097.	1062.91	{920., 1140.}	5.3938	0.0247864	Reff	0.778294	0.803221	{0.789213, 0.816772}	3.21935	ar	9240.	8236.28	{7281., 8538.}	10.8628
38	R0	{2.4454, 8.33501, 0.1}	{2.14455, 6.47945, 0.2}	{{2.14326, 6.48739, 0.}, {2.14326, 6.48739, 1.}}	{12.3027, 22.2622, 274.}	inf	1846.	1432.41	{1345.26, 1496.66}	22.4044	0.110823	newly	1286.	1257.98	{1199., 1313.}	3.26439	0.0144546	Reff	0.652922	0.661026	{0.648152, 0.673859}	1.57127	ar	8853.	8317.06	{8188., 8426.}	6.05377
39	R0	{2.9154, 12.3861, 0.1}	{2.73194, 11.2247, 0.97}	{{2.49869, 9.80052, 1.}, {2.80569, 11.6897, 1.}}	{6.29288, 9.37672, 876.}	inf	2561.	2329.05	{2028.9, 2475.63}	9.05685	0.0446434	newly	1272.	1269.37	{1221., 1315.}	2.40959	0.0106235	Reff	0.603196	0.624333	{0.603844, 0.647263}	3.57888	ar	9371.	9166.32	{8926., 9298.}	2.18419
40	R0	{2.40923, 15.1963, 0.1}	{2.04871, 11.6322, 3.}	{{2.04871, 11.6322, 3.}, {2.04871, 11.6322, 3.}}	{14.9643, 23.4536, 2900.}	inf	2019.	1718.56	{1675.51, 1760.41}	14.8807	0.0700511	newly	887.	966.74	{932., 1002.}	8.98985	0.0372305	Reff	0.893824	0.821621	{0.812826, 0.830308}	8.07798	ar	8735.	8206.17	{8123., 8287.}	6.05415
41	R0	{2.63137, 15.238, 1.}	{1.88486, 8.39294, 1.}	{{1.88486, 8.39294, 1.}, {1.88486, 8.39294, 1.}}	{28.3697, 44.921, 0.}	inf	2300.	1211.37	{1163.91, 1262.94}	47.3318	0.278685	newly	964.	908.06	{863., 949.}	5.98963	0.0270982	Reff	0.798621	0.777246	{0.767201, 0.786122}	2.67654	ar	9150.	7641.97	{7542., 7747.}	16.4812
42	R0	{2.48769, 19.4593, 0.1}	{1.6983, 8.21822, 1.03}	{{1.67405, 7.28757, 1.}, {1.71337, 8.80249, 1.}}	{31.7317, 57.7671, 930.}	inf	2205.	863.063	{753.516, 949.832}	60.8588	0.409161	newly	727.	653.65	{609., 690.}	10.1362	0.0468703	Reff	0.77392	0.75611	{0.747978, 0.764658}	2.30131	ar	8932.	6976.	{6745., 7177.}	21.8988
43	R0	{2.61199, 10.214, 0.1}	{2.68938, 10.3414, 0.04}	{{2.70795, 10.355, 0.}, {2.74056, 10.6768, 0.}}	{5.34501, 5.19839, 134.}	inf	2530.	2565.17	{2541.31, 2685.64}	5.87796	0.0277996	newly	1603.	1589.16	{1532., 1644.}	2.46413	0.0108796	Reff	0.846024	0.85776	{0.837026, 0.875022}	1.83283	ar	9061.	9123.09	{9101., 9248.}	1.90145
44	R0	{2.50994, 18.3382, 0.1}	{1.76803, 9.66766, 0.82}	{{1.71337, 8.80249, 0.}, {2.01705, 13.609, 1.}}	{29.5588, 47.2813, 756.}	inf	2193.	1095.92	{915.964, 1700.45}	50.0263	0.313347	newly	810.	734.44	{675., 836.}	10.879	0.0503914	Reff	0.937463	0.850991	{0.842755, 0.858828}	9.22403	ar	8827.	7192.73	{6842., 8157.}	18.5144
45	R0	{2.44283, 16.8338, 0.1}	{2.29457, 14.6129, 2.}	{{2.29887, 14.662, 2.}, {2.29887, 14.662, 2.}}	{6.06922, 13.1929, 1900.}	inf	2140.	1980.13	{1941.77, 2036.45}	7.47072	0.0342509	newly	873.	898.07	{862., 936.}	3.65521	0.0155949	Reff	0.849616	0.8344	{0.823247, 0.844648}	1.79604	ar	8878.	8623.27	{8558., 8716.}	2.86923
46	R0	{2.51815, 18.1462, 5.}	{2.45959, 17.5615, 1.08}	{{2.49092, 17.9391, 1.}, {2.49092, 17.9391, 1.}}	{2.32535, 3.22196, 78.4}	inf	2349.	2421.51	{2428.43, 2529.17}	7.89004	0.0390829	newly	861.	922.13	{889., 969.}	8.35889	0.0351116	Reff	0.909556	0.865926	{0.853568, 0.876691}	4.79685	ar	8978.	8900.2	{8931., 9035.}	1.26999
47	R0	{2.88885, 16.8987, 0.1}	{1.93419, 8.56054, 0.}	{{1.93419, 8.56054, 0.}, {1.93419, 8.56054, 0.}}	{33.0464, 49.342, 100.}	inf	2783.	1369.32	{1314.36, 1425.82}	50.7969	0.308204	newly	1104.	1040.02	{989., 1090.}	5.8279	0.0263413	Reff	0.890344	0.869549	{0.858235, 0.880023}	2.33553	ar	9337.	7766.56	{7662., 7884.}	16.8195
48	R0	{2.58709, 13.8786, 0.1}	{2.36315, 11.6509, 1.02}	{{2.14754, 9.98575, 1.}, {2.4534, 12.3139, 1.}}	{8.73397, 16.1881, 920.}	inf	2523.	2096.6	{1702.73, 2296.46}	17.0005	0.0831356	newly	1180.	1187.97	{1114., 1241.}	3.42627	0.0148619	Reff	0.854775	0.879259	{0.864365, 0.893344}	2.87025	ar	9146.	8704.24	{8278., 8910.}	4.84015
49	R0	{2.42959, 10.7132, 0.1}	{2.7042, 12.6293, 0.94}	{{2.71225, 12.7047, 1.}, {2.71225, 12.7047, 1.}}	{11.3576, 17.9961, 854.}	inf	2209.	2571.7	{2518.88, 2640.42}	16.503	0.066254	newly	1386.	1341.83	{1295., 1389.}	3.54329	0.0157501	Reff	0.872223	0.872557	{0.855831, 0.886765}	1.09679	ar	8863.	9154.86	{9094., 9205.}	3.30678
50	R0	{2.60513, 13.2796, 0.1}	{2.55018, 13.4971, 0.06}	{{1.74092, 5.83416, 0.}, {2.7499, 15.3124, 0.}}	{10.3335, 21.0177, 156.}	inf	2476.	2294.79	{1118.96, 2616.94}	13.1925	0.0729936	newly	1231.	1161.62	{1117., 1212.}	5.76442	0.0259923	Reff	0.977184	0.984989	{0.915298, 1.01676}	3.33289	ar	9095.	8821.21	{7217., 9217.}	4.21517
